# Powerful Antiseptic

**Published:** 1890-09

**Authors:** 


POWERFUL ANTISEPTIC.—Potassium mercuro-cyanide ($KHgCy_4$) is one of the most powerful antiseptics known. Behring found that 1 part in 60,000 will entirely prevent the development of the anthrax bacillus in serum. It is soluble in water and does not precipitate albumen. The lethal dose for guinea-pigs is 1–150,000 of the weight.

Sulphur, always heretofore considered an elemental substance, is now declared to be a compound of carbon with other elements. Dr. Theodore Gross, of Berlin, read a paper before the Vienna Academy of Sciences, detailing experiments which seemed to prove that sulphur, especially precipitated sulphur, and that is what is known as the allotropic form, is readily decomposed and leaves a residue of carbon.

